# Fertility preservation in gynaecologic malignancy: imaging role in treatment planning

**DOI:** 10.1186/1470-7330-15-S1-P35

**Published:** 2015-10-02

**Authors:** I Papadopoulou, M Qureshi, N Butterfield, N Bharwani, A Rockall

**Affiliations:** 1 Imperial College London, Exhibition Road, London, SW7 2AZ, UK

## Learning objectives

To know the options for fertility preservation in women with cervix, ovarian or endometrial cancer.

To understand the role and limitations of imaging in selection of patients for fertility preserving options.

To recognise the appearances of early stage disease that fulfil the criteria for eligibility for fertility preservation.

## Content organisation

A significant number of women with gynaecological cancer are of childbearing age and have not completed their families. In these younger women, consideration of fertility-sparing options is very important. This poster will describe the fertility-sparing treatment options that are currently available and delineate the role of imaging in the selection of suitable patients. A review of the current literature will be presented to provide an evidence base. In cervix cancer, MRI is used to delineate the size, position and stage of the tumour for the selection of patients suitable for radical trachelectomy. In complex adnexal masses, MRI with diffusion and perfusion is used to categorise the likelihood of invasive or borderline malignancy in order to allow potential fertility preservation where possible. In endometrial cancer, MRI is used to rule out imaging signs of invasive disease prior to consideration of hormonal treatment. Imaging is also used in patient follow-up for the detection of recurrent disease. Each of these scenarios will be illustrated.

## Conclusion

Imaging plays a major role in the management of patients who are considering fertility-preserving treatment options for gynaecologic malignancy.

